# Neurotrophic factors and neuroplasticity pathways in the pathophysiology and treatment of depression

**DOI:** 10.1007/s00213-018-4950-4

**Published:** 2018-06-30

**Authors:** Marion J. F. Levy, Fabien Boulle, Harry W. Steinbusch, Daniël L. A. van den Hove, Gunter Kenis, Laurence Lanfumey

**Affiliations:** 1 Centre de Psychiatrie et Neurosciences (Inserm U894), Université Paris Descartes, 102-108 rue de la santé, 75014 Paris, France; 2 Department of Psychiatry and Neuropsychology, Maastricht University, Maastricht, The Netherlands; 3 EURON—European Graduate School of Neuroscience, Maastricht, The Netherlands; 4 Department of Psychiatry, Psychosomatics and Psychotherapy, University of Wuerzburg, Wuerzburg, Germany

**Keywords:** Growth factors, Neurocircuits, Plasticity, Antidepressant, Mood

## Abstract

Depression is a major health problem with a high prevalence and a heavy socioeconomic burden in western societies. It is associated with atrophy and impaired functioning of cortico-limbic regions involved in mood and emotion regulation. It has been suggested that alterations in neurotrophins underlie impaired neuroplasticity, which may be causally related to the development and course of depression. Accordingly, mounting evidence suggests that antidepressant treatment may exert its beneficial effects by enhancing trophic signaling on neuronal and synaptic plasticity. However, current antidepressants still show a delayed onset of action, as well as lack of efficacy. Hence, a deeper understanding of the molecular and cellular mechanisms involved in the pathophysiology of depression, as well as in the action of antidepressants, might provide further insight to drive the development of novel fast-acting and more effective therapies. Here, we summarize the current literature on the involvement of neurotrophic factors in the pathophysiology and treatment of depression. Further, we advocate that future development of antidepressants should be based on the neurotrophin theory.

## Introduction

Depression has emerged over the past decades as a major debilitating disease with a high prevalence in occidental populations, resulting in profound social and economic burden (Lopez and Murray 1998; Nestler et al. 2002; Pincus and Pettit 2001; Wittchen et al. 2011). Despite recent advances in neuroscience research, the neurobiological mechanisms underlying the pathophysiology of depression remain poorly understood. The development and course of major depressive disorder (MDD) are likely to be mediated by a complex interaction between genetic and environmental factors, and the associated heterogeneity of the disease makes it difficult to develop effective therapeutic treatments (Keers and Uher 2012). So far, many classes of antidepressants have been discovered and marketed for the treatment of depression. However, currently available antidepressants display significant limitations, including a delayed onset of action, low response rates, and relapse after treatment cessation, which remain major drawbacks for a disease with relatively high suicide rates (Angst et al. 2002). To date, clinical and preclinical studies have linked depression to structural and cellular alterations, such as neuronal loss and synaptic dysfunction, in cortico-limbic brain regions controlling mood and emotions (Duman and Aghajanian 2012). Among many candidates, neurotrophic growth factors and related signaling pathways constitute major players in neuroplasticity, and current evidence indicates that impairment in growth factor signaling is associated with depressed mood (Pittenger and Duman 2008). Interestingly, currently prescribed antidepressants have been shown to increase neuroplasticity when exerting their therapeutic effects (Tardito et al. 2006). Hence, a deeper understanding of the exact molecular, cellular, and structural plasticity mechanisms involved in antidepressant action might lead to the identification of key effectors and provide further insight into the development of novel fast-acting and more effective therapies. Here, we summarize the current literature on the implication of neurotrophic factors and associated signaling pathways in depression and antidepressant treatments. First, a brief overview of the brain regions and circuits implicated in the pathophysiology of depression and in the response to antidepressants is highlighted. Further, evidence for the involvement of neurotrophic factors and associated signaling pathways in depression and its current treatment is described. Finally, a perspective towards the development of novel antidepressant drugs is given.

## Brain regions and neurocircuits involved in depression: neuroanatomical evidence

### Amygdala

The amygdala is an integrant part of the limbic system implicated in cognitive and emotional processing, in particular that involved in fear and anxiety (Aggleton 1993; LeDoux 2000). This infers to this structure a central role in the regulation of emotion and in consequence, in mood-related pathology. Although volumetric magnetic resonance imaging (MRI) studies so far revealed contrasting results, with studies showing either an increase (Frodl et al. 2003; Lange and Irle 2004; Vassilopoulou et al. 2013) or a decrease in amygdalar volume in depressed patients (Bellani et al. 2011; Kronenberg et al. 2009; Lorenzetti et al. 2009), most of the functional MRI (fMRI) studies showed an increased activity of the amygdala in depressed patients during encoding of negative but not neutral or positive stimuli. Indeed, studies in depressed patients have shown exaggerated left and right amygdala activity when confronted with emotional facial expressions (Canli et al. 2005; Peluso et al. 2009; Sheline et al. 2001). Similarly, Drevets et al. using positron emission tomography (PET) imaging reported an increase in amygdala activation and metabolism in MDD patients (Drevets 2003). The fact that higher amygdala activity was often observed after negative stimuli would explain the higher ability for depressed individuals to encode and remember negative rather than positive information, therefore contributing to the negative bias observed in depressed patients (Groenewold et al. 2013; Hamilton and Gotlib 2008).

Interestingly, in studies using diffusion tensor imaging or MRI, abnormal microstructure and connectivity of the amygdala and the medial prefrontal cortex (PFC) patients (Arnold et al. 2012) as well as reduced functional coupling between the amygdala and the supragenual PFC (Matthews et al. 2008) were reported in remitted MDD. These findings suggest that MDD might result, at least in part, from a failed ability to co-activate a top-down cognitive control network during emotion processing (Matthews et al. 2008). This is supported by data from Pezawas et al. (2005) who reported that, in individuals carrying the short (s) allele of a variable number of tandem repeats in the 5′ promoter region of the serotonin transporter gene (5-HTTLPR), increased anxiety—and consequently an increased risk for depression—was associated with a reduced amygdala–anterior cingulate cortex connectivity. These data further support the hypothesis of the alteration of the negative feedback from the PFC to the amygdala in depressed patients (Pezawas et al. 2005).

In rodent, while a decrease in basolateral amygdalar volume has been associated with an increase in both fear and stress reactivity in mice (Yang et al. 2008), an enhanced dendritic arborization, elongation, and spine density, providing evidence for increased synaptic connectivity within the amygdala, were reported after chronic stress exposure in rats (Vyas et al. 2006; Vyas et al. 2002). These data were in line with those showing an enhanced activity of the basolateral amygdala and long-lasting anxiety-like responses in rats that received repeated injections of urocortin, an agonist of corticotropin releasing factor (CRF) receptors (Rainnie et al. 2004). This further suggests that depression-like behavior is associated with increased amygdala activity.

Interestingly, antidepressants have been shown to normalize most of these defects. Indeed, a meta-analysis from MRI studies showed that amygdalar volume was increased in medicated patients (Hamilton et al. 2008). Given that amygdala hyperactivity could lead to the amygdalar volume reduction observed in depression (Siegle et al. 2003), it could therefore be expected that antidepressants would decrease amygdala activity as well. Indeed, several meta-analyses concluded that antidepressants facilitate positive emotional stimuli processing in MDD patients and reduce the activity of negative emotions (Delaveau et al. 2011), concomitant with a normalization of amygdala activity (Chen et al. 2014; Victor et al. 2010).

### Hippocampus

The hippocampus is also a major structure within the limbic system known to be highly vulnerable to stress and other environmental factors. This region is critical in diverse cognitive processes and in the regulation of emotions (Bartsch and Wulff 2015). MRI analyses revealed reduced hippocampal volume in patients suffering from both first episode and recurrent depression (Bremner et al. 2000; Cole et al. 2011; Frodl et al. 2007). Besides, a correlation between volume reductions and total duration of major depression has been reported more than 20 years ago (Sheline et al. 1996). These results have been further confirmed by several meta-analyses that show, e.g., a decreased hippocampal volume only in MDD patients having suffered from depression for 2 years or who had more than one episode (McKinnon et al. 2009) or evidencing deficit in hippocampal volume deficits in recurrent but not in first episode MDD patients (Schmaal et al. 2016), although this last study gave rise to some debate (Fried and Kievit 2016). It has also been proposed that reductions in hippocampal volume may not antedate illness onset but that hippocampal volume may decrease most in the early years after illness onset (MacQueen et al. 2003). Post-mortem analysis in MDD patients suggested an increase in the density of pyramidal, granule, and glial cells combined with a decrease of soma size of pyramidal cells (Stockmeier et al. 2004). This could indicate that the decrease in cellular neuropil might account for the reduced hippocampal volume found in depressed subjects.

Furthermore, disrupted functional hippocampal connectivity within the prefrontal and parietal cortex has been revealed by fMRI in MDD patients (Cao et al. 2012; Delaveau et al. 2011; Jaworska et al. 2015; Milne et al. 2012; Toki et al. 2014).

Animal studies revealed that chronic stress causes atrophy of apical dendrites of pyramidal neurons in the CA3 region of the hippocampus (Magarinos et al. 1996; Vyas et al. 2002; Watanabe et al. 1992). Exposure to excess glucocorticoids in rats showed decreased apical branching numbers and apical dendrite length (Woolley et al. 1990), suggesting a role for the activation of the hypothalamo-pituitary-adrenal (HPA) axis in remodeling hippocampal morphology. A wealth of data have clearly evidenced a correlation between hyperactivity of the HPA axis and the development of depression (Frodl and O’Keane 2013). Indeed, HPA axis activation is mainly triggered by stress, which has been shown to be strongly involved in inducing depression (Kendler et al. 1995). In addition, various models of chronic stress exposure in rodents have indicated a decrease in neurogenesis in the dentate gyrus (DG) of the hippocampus, involving a reduced proliferation, survival, and differentiation of neural stem cells (Eisch and Petrik 2012).

When treated with antidepressants (Boldrini et al. 2013; Fu et al. 2013; Sheline et al. 2003) or electroconvulsive therapy (ECT) (Nordanskog et al. 2014), depressed patients showed increased hippocampal volume. Interestingly, a meta-analysis reported that antidepressants could decrease the hypersensitivity to negative stimuli by decreasing hippocampus hyperactivation (Delaveau et al. 2011). Similarly, antidepressant such as imipramine could restore the total number of cells in the hippocampus impaired by social defeat stress in mice (Van Bokhoven et al. 2011). In addition, the same antidepressant could increase the number of hippocampal neurons in Flinders Sensitive Line rats, a genetic rat model of depression that shows impaired cell proliferation (Chen et al. 2010).

Altogether, these findings provide further evidence of the crucial role of the hippocampus in depression.

### Prefrontal cortex

The PFC is functionally connected with several brain structures, for processing sensory input and mediating executive motor functions. The ventromedial PFC and the orbitofrontal cortex are involved in the cognitive processing of emotional stimuli originating from the limbic system (e.g., amygdala, ventral striatum, hippocampus, and hypothalamus) and are especially engaged in memory consolidation and retrieval (Ongur and Price 2000; Price 1999). As such, the PFC plays a major role in regulating the appropriate emotional response such as fear or anxiety. Moreover, the PFC has been associated with decision-making, personality expression, social behavior, and hedonic responses (Mitterschiffthaler et al. 2003).

Neuroimaging studies showed a reduction in size of multiple areas of the PFC in subjects diagnosed with MDD (Bremner et al. 2002; Drevets 2000). In line with those studies, post-mortem brain analysis of depressed patients revealed reduced neural cell size and neural and glial cell densities as well as synapse number in the dorsolateral and subgenual PFC (Cotter et al. 2002; Kang et al. 2012; Öngür et al. 1998; Rajkowska et al. 1999). The PFC is strongly connected with the amygdala and the hippocampus and the activity of its different subdivisions has been widely studied in depressed patients. Hence, although studies seemed consistent in showing a lower activity of dorsolateral PFC in resting state analyses of MDD patients (Fitzgerald et al. 2008; Hamilton et al. 2012; Limon et al. 2016; Zhang et al. 2015a; Zhong et al. 2016), meta-analyses of this region during task processing and especially in response to emotional stimuli with a negative valence have shown either a higher activity (Miller et al. 2015; Wang et al. 2015b; Zhang et al. 2013) or a lower activity (Groenewold et al. 2013; Hamilton et al. 2012; Zhang et al. 2015a). This might be due to the various parameters of the studies such as age of individuals, severity of depression, or whether there were medicated or not. The last point corroborates the hypothesis of impaired executive functions leading to emotional biases and dysregulation in MDD. Furthermore, the study of other PFC subregions tended to show a hyperactivity of the ventrolateral, the orbitofrontal, and ventromedial PFC in MDD patients (Groenewold et al. 2013; Limon et al. 2016; Miller et al. 2015).

Regarding preclinical studies, chronic restraint stress caused a significant reduction in the number and length of apical dendritic branches of pyramidal neurons in PFC areas (Cook and Wellman 2004). Similar results were observed in rats that received chronic administration of corticosterone. In this rodent model for stress-related disorders, a drastic dendritic reorganization of pyramidal neurons was also reported in the medial PFC (Wellman 2001). Furthermore, in rats expressing a depressive-like behavior, cell activity measured by c-Fos immunoreactivity was decreased in the ventromedial PFC (Lim et al. 2015). These data were supported by a study showing a decrease in both the excitatory and inhibitory neuronal functions in the PFC and a disturbed neurotransmitter homeostasis in the social defeat mouse model of depression (Veeraiah et al. 2014).

PFC abnormalities observed either in MDD patients or in rodent models are partly corrected by antidepressants. An 8-week escitalopram treatment was shown to reduce irregular high functional connectivity in the bilateral dorsal medial PFC in MDD patients (Lyttle et al. 2015; Wang et al. 2015a) and, in rats, a 2-week administration of a selective serotonin reuptake inhibitor (SSRI), fluvoxamine, restored dendritic length and spine densities but not cortical thickness after early-life stress exposure (Lyttle et al. 2015). Furthermore, it appeared that both high- and low-frequency electrical ventromedial PFC stimulations in rat models of depression attenuated depressive-like behavior (Bruchim-Samuel et al. 2016; Lim et al. 2015).

### Ventral striatum

Finally, a preponderant role for the ventral striatum has also been reported in major depression. The fundamentals of the natural reward system are attributed to the dopaminergic connections between the ventral tegmental area (VTA) and the nucleus accumbens (NAc). In this respect, the NAc and the VTA may play a role in mediating the anhedonic symptoms of depression (Nestler and Carlezon Jr. 2006; Russo and Nestler 2013; Yadid and Friedman 2008). Depressed patients show an attenuated activation of the VTA-NAc pathway or the NAc itself when compared to normal patients in fMRI analyses (Epstein et al. 2006; Furman et al. 2011; Pizzagalli et al. 2009; Smoski et al. 2009). In rodent models of depression, a reduced dopaminergic activity in the NAc (Shirayama and Chaki 2006) with a disturbed burst firing of VTA neurons was also observed (Friedman et al. 2008).

Deep brain stimulation (DBS) of the NAc has been shown to influence the functionality of efferent projections of the NAc to the hippocampus (Settell et al. 2017). DBS targeting the NAc was shown to exert antidepressant, anxiolytic, and hedonic effects, notably in treatment-resistant depression (Bewernick et al. 2010; Giacobbe et al. 2009; Schlaepfer et al. 2008). These effects have been proven to be long-lasting and stable for up to 4 years (Bewernick et al. 2012; Malone Jr. et al. 2009). While MDD patients demonstrated reduced ventral striatal activation during anticipation of gain and loss, a treatment with the SSRI escitalopram was able to normalize this hyporesponsiveness (Stoy et al. 2012). Preclinical studies also showed that, in response to NAc DBS, more neuronal precursors were found in the dentate gyrus of the hippocampus, hinting at enhanced adult neurogenesis (Schmuckermair et al. 2013). NAc DBS may also alter the morphology of the PFC, with increases in apical and basilar dendrite length (Falowski et al. 2011).

**Fig. 1:**
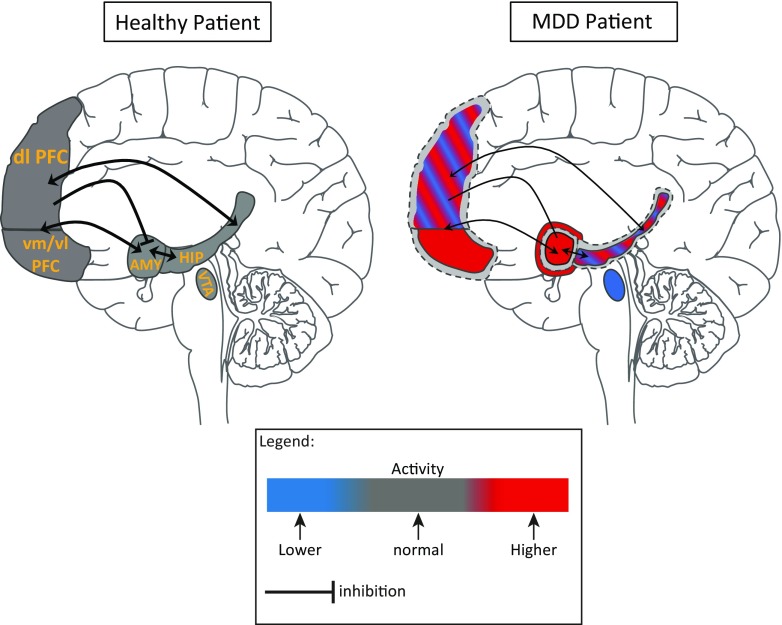
Summary of the neuroanatomical changes observed in MDD patients. In the MDD brain, the dotted lines correspond to the brain volume in a healthy patient. In the HIP and the dlPFC, the alternative blue and red colors show the discrepancies reported in the different studies regarding their activities in MDD patients. The thinner arrows and lines show reduced connectivity between the regions. dlPFC, dorsolateral prefrontal cortex; vm/vlPFC, ventromedial/ventrolateral prefrontal cortex; HIP, hippocampus; AMY, amygdala; VTA, ventral tegmental area. See text for more details. This illustration was taken from “Servier medical art” (http://www.servier.fr/servier-medical-art)

Overall, despite few discrepant findings, studies so far suggest that MDD is associated with structural and functional changes in brain regions, especially those described above, and alterations in their mutual connectivity (for summary, see Fig. 1). These changes are the likely result of alterations in neuroplastic processes that regulate synaptic connectivity and maintain neuronal integrity. Understanding the nature and molecular underpinnings—including neurotrophic signaling—of these processes may provide new clues for succesful pharmacological interventions. The next sections explore alterations of neuroplasticity in the context of MDD.

## Neuroplasticity changes in MDD and the effects of antidepressant therapies

### Neuroplasticity: definition

Neuroplasticity can be defined as the ability of the nervous system to respond to intrinsic or extrinsic stimuli by reorganizing its structure, function, and connections (Cramer et al. 2011). It includes different mechanisms as described in an excellent review by Castren and Hen (2013). One of them is neurogenesis, the formation of newborn neurons in proliferative areas. The regions identified so far in the rodent adult brain are the subventricular zone (SVZ) and the subgranular zone (SGZ) of the DG in the hippocampus. Another mechanism of plasticity is the modification of mature neuronal morphology, involving axonal and dendritic arborization and pruning, an increase in spine density, and synaptogenesis. At a functional level, long-term potentiation (LTP) is the main mechanism mediating plasticity. The transcriptional regulation of genes involved in neuroplasticity by epigenetic mechanisms also contributes to synaptic plasticity. Altogether, these processes mediated the dynamic and adaptive changes in synaptic strength (Castren and Hen 2013).

### Neuroplasticity changes in MDD

While neuroplasticity in rodents has been well documented during the last decades, the study of neuroplasticity in the human brain largely remains indirect, mostly because of methodological limitations as well as ethical constraints.

However, some alternative methods such as the assessment of the hippocampal DG volume by MRI, magnetic resonance spectroscopy (Bergmann et al. 2015; Ho et al. 2013), and more recently the use of ^14^C in genomic DNA labeling (Spalding et al. 2013) have provided some valuable information on human neuroplasticity. Most of the post-mortem studies have now evidenced that in adult humans, new neurons continued to be generated with a modest decline during aging (Eriksson et al. 1998; Reif et al. 2006; Spalding et al. 2013). Although recently questioned by Sorrells et al. (2018) who suggested that human neuroplasticity could differ from other species, it has been nevertheless confirmed that in human DG, about 700 new neurons were generated per day whatever the age (Boldrini et al. 2018).

Beside neurogenesis, it is also rather well-established that LTP can be induced in the human CNS with similar molecular mechanisms than those observed in rodent models (Bliss and Cooke 2011). Human LTP has first been demonstrated in isolated cortical tissue obtained from patients undergoing surgery, and recent studies have shown that LTP-like phenomena can be obtained in the human cortex as well by using repetitive presentations of sensory stimuli while recording event-related potentials from the scalp (Clapp et al. 2005a, b).

In MDD, neuroimaging and post-mortem studies in humans indicate that structural changes are often observed in the course of this pathology. Structural MRI studies have revealed reduced hippocampal volume in individuals during a depressive episode in comparison to patients in remission (Kempton et al. 2011), while increased hippocampal dendritic atrophy and cell death as well as reduced LTP and BDNF expression have also been reported (Miller and Hen 2015; Pittenger 2013). While MRI studies were rather consistent with the observation of a reduced DG size in patients with depression or anxiety disorders (Boldrini et al. 2013; Bremner et al. 2000; Cole et al. 2011; Frodl et al. 2007; Huang et al. 2013), post-mortem studies in depressed patients showed important disparities regarding neurogenesis, showing either no difference (Reif et al. 2006) or a decrease in the number of DG progenitor cells (Lucassen et al. 2010). Although these findings make it tempting to speculate on reduced levels of neurogenesis in MDD, further investigations making use of more specific techniques are needed to better understand the dynamics of adult neurogenesis in MDD. However, other attempts to measure human brain plasticity in the course of MDD have demonstrated functional alterations such as those observed at LTP level. For example, visual-evoked potential amplitudes in the visual pathway were, compared to matched control subjects, decreased in patients with depression (Normann et al. 2007; Bubl et al. 2015) and also in bipolar disorder patients (Elvsashagen et al. 2012).

In preclinical depression-like models as well, consistent data have reported a decrease in the proliferation and survival of hippocampal neurons when the HPA axis was dysregulated. Hence, using proliferation and survival cell markers such as BrdU, Ki-67, or DCX, impaired neurogenesis was observed in rodent models of depressive-like behavior. These models included transgenic mice (Paizanis et al. 2010), corticosterone-induced mouse model of depression/anxiety (Zhang et al. 2014b), or rats subjected to chronic mild stress (Morais et al. 2017 and see Anacker and Hen 2017 for a review). Beside neurogenesis, numerous studies have also reported that synaptic plasticity was also greatly impaired in stress models of depression (Pittenger 2013). Severe stress has also been shown to inhibit LTP (Kim and Diamond 2002) and enhance LTD (Xu et al. 1997) in the hippocampus and in prefrontal pyramidal cells (Goldwater et al. 2009).

Altogether, these data strongly suggest synaptic plasticity is strongly affected in MDD, at both structural and functional levels, and that these alterations are similar to those evidenced in rodent studies.

### Effects of antidepressant therapies on plasticity

#### Electroconvulsive therapy

Recent clinical studies showed that electroconvulsive therapy (ECT) promoted structural plasticity including increased volume and morphometric changes in the hippocampus and the amygdala along with improved clinical responses, especially in patients with a smaller hippocampal volume (Joshi et al. 2016; Nordanskog et al. 2010; Tendulkar et al. 2013). ECT may also reduce cortical excitability and, thereby reverse increases in the excitability of cerebral cortex, during treatment-resistant depression (Sackeim et al. 1983). In addition, low-frequency trains of transcranial magnetic stimulation (rTMS) applied on several regions of the brain to induce LTP- and LTD-like changes in neuronal activity produced identical effects to those achieved with ECT in depressive patients (Fitzgerald and Daskalakis 2011).

Similar results have been demonstrated in rodent models of depression (Nakamura et al. 2013; Schloesser et al. 2015) and non-human primates (Perera et al. 2007).

#### Exercise

Exercise has also shown beneficial effects on plasticity. Although it has been proven that exercise in MDD patients reduced depressive symptoms (Herring et al. 2012; Ota and Duman 2013; Schuch et al. 2016), neuroplasticity per se has not yet been monitored in patients in these conditions, due to the limitations mentioned in section “Neuroplasticity changes in MDD.” However, a meta-analysis has suggested that acute aerobic, but not strength exercise, increases basal peripheral BDNF concentrations, although this effect was only transient (Knaepen et al. 2010). A recent study showed an increase in synchronous neuronal responses during a task that requires an upregulation of cognitive control when exercise was combined with meditation (Alderman et al. 2016). In preclinical studies, swimming showed antidepressant-like effects on anhedonia in stressed rodents along with a normalization of a stress-induced BDNF mRNA expression decrease (Jiang et al. 2014). A 21-day exercise regimen in rats transiently increased LTP in the DG of the hippocampus (Radahmadi et al. 2016), although recent data suggested that, if motor activity could exert positive effects on cognitive processes, it was under very controlled conditions (D’Arcangelo et al. 2017) and that acute swim stress could also led to LTD (Tabassum and Frey 2013).

#### Antidepressant treatments

Few studies have directly addressed the effects of antidepressant therapy on neuroplasticity in the human brain. Imaging studies have provided data showing that, e.g., in MDD patients taking medication for 3 years, the left hippocampus increased in volume compared to the beginning of the study (Frodl et al. 2008). Interestingly, the volume of the left hippocampus has also been shown to enlarge under lithium treatment in elderly bipolar patients, probably through a neuroprotective effect (Zung et al. 2016).

Regarding neurogenesis per se, the analysis of post-mortem brains of MDD patients treated with nortriptyline and clomipramine showed an increase of neural progenitor cells and dividing cells in the DG, as compared to healthy controls (Boldrini et al. 2009). In addition, the same group reported that both the fewer mature granule neurons and the smaller DG and granule cell layer volume found in post-mortem brain tissue of depressive patients were reversed by antidepressant treatment (Boldrini et al. 2013). Interestingly, a meta-analysis concluded that depressed patients with a decreased hippocampal volume showed lower response/remission rates after antidepressant treatment (Colle et al. 2016).

In a rodent model of depression, both reduced hippocampal proliferation and increased cell death were reversed by chronic administration of antidepressants (Pilar-Cuellar et al. 2013). Adult hippocampal neurogenesis was proposed to be required for the therapeutic action of antidepressants (David et al. 2009; Djavadian 2004; Klempin et al. 2013; Sahay and Hen 2007; Santarelli et al. 2003). Accordingly, chronic treatment with a SSRI such as fluoxetine increased hippocampal neurogenesis through the generation of newborn cells in the DG (Boldrini et al. 2009; Encinas et al. 2006; Malberg et al. 2000; Santarelli et al. 2003; Surget et al. 2011). In addition, serotonin and noradrenaline reuptake inhibitor (SNRI) antidepressants, like SSRIs, also modulate neurogenesis and plasticity. Neurogenesis in the DG of the hippocampus was increased following chronic venlafaxine administration to rats (Mostany et al. 2008). Similarly, chronic venlafaxine treatment proved to be efficient in preventing the deleterious effects of restraint stress on hippocampal neurogenesis and BDNF protein expression (Xu et al. 2006). Likewise, tricyclic antidepressants (TCAs) have also been shown to modulate hippocampal neurogenesis. Clomipramine was able to counteract the stress-induced inhibition of proliferation in the hippocampus (Liu et al. 2008). Chronic imipramine and desipramine treatment increased cell proliferation in the SGZ (Pechnick et al. 2011; Santarelli et al. 2003; Schiavon et al. 2010).

In addition to their effects on neurogenesis, evidence has also been generated that antidepressants can regulate other types of plasticity. Treatments with classical antidepressants are indeed able to modulate LTP within the hippocampus (Wang et al. 2008) and chronic fluoxetine administration has also been demonstrated to increase synaptic plasticity in naive rats (Stewart and Reid 2000).

Overall, these data showed that antidepressants have a rather beneficial effect on neuroplasticity. However, they still show lack of efficacy in some depressed patients. Indeed, MDD is a complex disorder of which the etiology remains unclear and that cannot be simplified by neuroplasticity dysfunctions as the only targetable factor.

## Neurotrophins and other growth factors

**Fig. 2:**
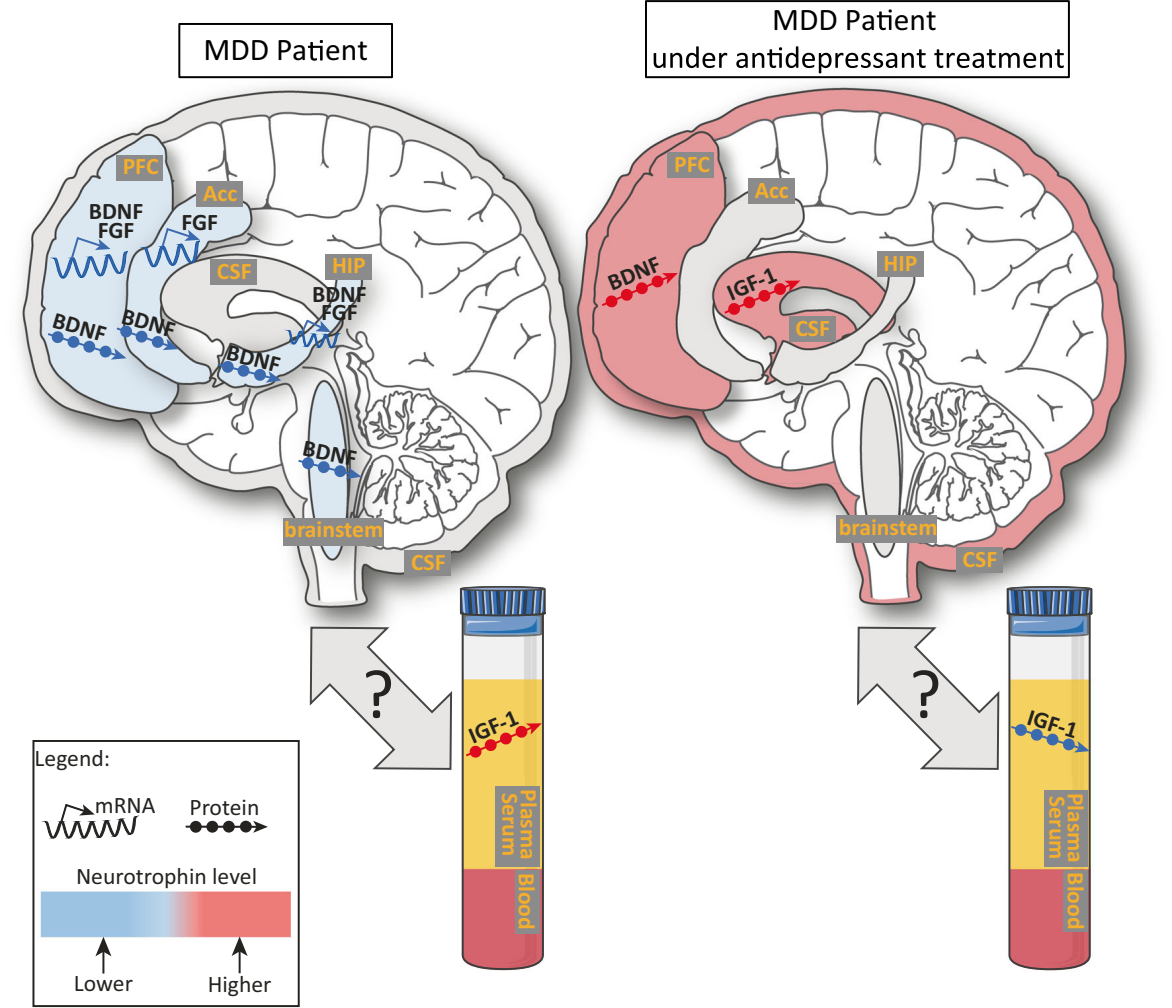
Neurotrophin level changes observed in MDD patients with or without antidepressant treatment. Observed changes in neurotrophin levels, in MDD patients whether or not under antidepressant treatment, represented in the different brain areas involved in depression, and in blood (IGF-1 only). The blue color corresponds to a low level of neurotrophin while the red color shows a high level. Arrows with the single strand represent RNA expression changes and arrows with circles protein level changes. PFC, prefrontal cortex; Acc, anterior cingulate cortex; c.brainstem, caudal brainstem; CSF, cerebrospinal fluid; HIP, hippocampus. See text for more details. This illustration was taken from “Servier medical art” (http://www.servier.fr/servier-medical-art)

Growth factors have been proven to be strongly involved in regulating plasticity by impacting upon almost all of the neuroplasticity processes mentioned in section “Neuroplasticity changes in MDD and the effects of antidepressant therapies,” including neuronal survival, differentiation, and proliferation (Crutcher 1986; Lu et al. 2014). Accordingly, this section focuses on the main studied growth factors that have been described thus far, from their general functions to their role in MDD. This is summarized in Table 1 and Fig. 2.

**Table 1:** Involvement of neurotrophins and growth factors in human depression and in animal models of depression

Neurotrophins and growth factors	Models	Structures	Changes	References	Treatments	Effects	References
Brain-derived neurotrophic factor (BDNF)	Post-mortem brain analysis of suicide victims or depressed patients	HIP	↓	Dwivedi et al. (2003), Pandey et al. (2008)	Antidepressants	↑	Chen et al. (2001), Dunham et al. (2009)
		PFC	↓	Dwivedi et al. (2003), Pandey et al. (2008)			
		Acc	↓	Youssef et al. (2018)			
		c.brainstem	↓	Youssef et al. (2018)			
	Rat models of depression	Brain			Chronic antidepressant, ECT	↑	Altar et al. (2003), Balu et al. (2008), Jacobsen and Mork (2004), Nibuya et al. (1995)
		PFC	↓	Zhang et al. (2015b)	Infusion of BDNF	Pro-depressive effect	Eisch et al. (2003)
		VTA-Nac	↑	Shirayama et al. (2015), Zhang et al. (2015b)	Infusion of BDNF	Pro-depressive effect	Eisch et al. (2003)
					Knockdown of BDNF	Antidepressant-like effect	Berton et al. (2006)
		HIP	↓	Zhang et al. (2015b)	Infusion of BDNF	Antidepressant-like effects	Deltheil et al. (2009), Shirayama et al. (2002), Sirianni et al. (2010)
					Knockdown of BDNF	Pro-depressive behavior	Taliaz et al. (2010)
Fibroblast growth factor (FGF)	Post-mortem brain analysis of depressed patients	dl PFC	↓	Evans et al. (2004)			
		Anterior cingulate cortex	↓	Evans et al. (2004)			
	Depressed patients	HIP	↓	Gaughran et al. (2006)			
	Rodent models of depression	HIP	↓	Turner et al. (2008a)			
	Rats	Lateral ventricle			Infusion of FGF	Antidepressant-like effect	Turner et al. (2008b)
Vascular endothelial growth factor (VEGF)	Rats with chronic stress	HIP	↓	Heine et al. (2005)			
	Rats				Antidepressant + VEGF	Antidepressant-like effect	Greene et al. (2009), Warner-Schmidt and Duman (2007)
Glial cell line-derived neurotrophic factor (GDNF)	Post-mortem brain analysis of depressed patients	Parietal cortex	↑	Michel et al. (2008)			
	Rats	C6 glioblastoma cell line			Antidepressant treatment	GDNF release	Hisaoka et al. (2001)
		PFC			Antidepressant treatment	↑	Angelucci et al. (2003b)
		HIP				↓	
Insulin-like growth factor (IGF-1)	Depressed patients	serum	↑	Bot et al. (2016), Kopczak et al. (2015), Szczesny et al. (2013)	Antidepressants	↓	Bot et al. (2016), Kopczak et al. (2015)
		Cerebrospinal fluid			Antidepressants	↑	Schilling et al. (2011)
	IGF-1 KO mice		Susceptibility to depression	Mitschelen et al. (2011)			
	Rodents				Infusion of IGF-1	Antidepressant effects	Duman et al. (2009), Hoshaw et al. (2005), Malberg et al. (2007), Park et al. (2011)
Nerve growth factor	Rat models of depression	HIP			ECT	↑ Antidepressant effect	Aarse et al. (2016), Angelucci et al. (2003a), Overstreet et al. (2010)
	Rat models of depression	HIP			NGF injections	↑ Antidepressant effect	Aarse et al. (2016). Angelucci et al. (2003a), Overstreet et al. (2010)

*MDD* major depressive disorders, *HIP* hippocampus, *PFC* prefrontal cortex, *Acc* anterior cingulate cortex, *c.brainstem* caudal brainstem, *ECT* electroconvulsive therapy, *VTA-NAc* ventral tegmental area-nucleus accumbens, *dl PFC* dorsolateral PFC, *SNRI* serotonin–norepinephrine reuptake inhibitor

### Brain-derived neurotrophic factor or BDNF

#### General function

BDNF is a neurotrophin involved in the growth, differentiation, and survival of neurons and has also been shown to represent an important factor in the regulation of neurogenesis and synaptic plasticity (Lu et al. 2014). It exerts its neurotrophic effects by activating the tropomyosin-related kinase receptor B (TrkB) (Chao and Hempstead 1995). It also binds, albeit with a lower affinity, to the p75^NTR^ receptor, which is generally known to promote proteolysis and apoptosis (Boulle et al. 2012). BDNF is abundantly expressed in the mammalian brain, with the highest concentrations found in the hippocampus and cortex (Ernfors et al. 1990).

#### BDNF and neuroplasticity

Several in vitro studies have been conducted in order to unravel the effects of BDNF on plasticity. Indeed, when PC12 cells transfected with TrkB were stimulated with BDNF for 48 h, neurite outgrowth was increased compared to the non-treated cells (Cazorla et al. 2011). Interestingly, in growth medium B27-deprived primary hippocampal cells, BDNF stimulation was able to promote dendritic outgrowth and spine formation (Park et al. 2016) and this neuroplastic effect is probably achieved through intracellular signaling cascades (Cavanaugh et al. 2001; Obrietan et al. 2002). As such, BNDF has been shown to increase the activity of the mitogen-activated protein kinase (MAPK) cascade promoting survival in neural cell cultures (Hetman et al. 2002).

In vivo evidences also support the critical role of BDNF in plasticity. In particular, mutation studies have demonstrated the role of this neurotrophin in structural and synaptic plasticity. Total BDNF deficiency is lethal and most of the mice lacking BDNF die during the second postnatal week (Ernfors et al. 1994). However, heterozygous BDNF knockout mice survive into adulthood and the use of these mice evidenced that BDNF was required for several forms of LTP (Aarse et al. 2016). This was in agreement with data that showed that BDNF infusion in the rat hippocampus induced LTP and triggered synaptic strengthening (Bramham 2007; Ying et al. 2002). At morphological level, these mice display a specific hippocampal volume reduction (Lee et al. 2002; Magarinos et al. 2011) similarly to what was found in heterozygous TrkB mice (von Bohlen und Halbach et al. 2003) but in contrast to p75^NTR^-deficient mice (Dokter et al. 2015), suggesting a link between hippocampal volume and BDNF-mediated TrkB signaling (von Bohlen und Halbach et al. 2003; von Bohlen Und Halbach and von Bohlen Und Halbach 2018). In addition, in the hippocampus, BDNF increases the total length, but not the branching, of apical dendrites within the CA1 *stratum radiatum*, without affecting basal dendrites in the *stratum oriens* (Alonso et al. 2004). However, in mutant mice in which *bdnf* excision mediated by Cre recombinase led to an almost total disappearance of BDNF in the brain, the volume of the hippocampus was mostly unchanged except for small changes in dendritic branches in restricted segments and signs of a modest delay in spine maturation (Rauskolb et al. 2010). Although these data and other (Baquet et al. 2004) indicate a temperate effect of BDNF in the maintenance of the cellular architecture of the adult brain, most of the studies evidenced its central role in neuronal plasticity.

#### BDNF in MDD

BDNF in MDD has been largely documented. However, many of the studies addressed peripheral (blood) BDNF concentration, with the assumption that blood BDNF could be a biomarker reflecting that of brain tissue (see, e.g., Klein et al. 2011). However, there is no evidence that serum BDNF is related to brain BDNF and neuroplasticity. The origin of serum BDNF has been now clearly documented and was demonstrated to come from the progenitors of platelets (Chacón-Fernández et al. 2016). Nevertheless, lowered serum concentrations of BDNF have often been associated with MDD (Karege et al. 2002, 2005; Sen et al. 2008, Molendijk et al. 2011). A thorough meta-analysis by Molendijk et al. showed that, despite demonstrable study heterogeneity, serum BDNF levels are overall lower in depressed patients (Molendijk et al. 2014). These findings were recently confirmed by two other meta-analytic efforts (Polyakova et al. 2015; Kishi et al. 2017). However, the significant variation between available assays (Polacchini et al. 2015) prevents the use of serum BDNF (and other neurotrophins) levels as a reliable biomarker for mood disorders.

Nevertheless, within the CNS, a reduction in BDNF and TrkB expression in the hippocampus and PFC has been reported in post-mortem brain tissue of suicide victims (Dwivedi et al. 2003; Pandey et al. 2008). One of the most common functional single nucleotide polymorphism (SNP) in the *Bdnf* gene is rs6265, causing a valine to methione substitution at codon 66 (val66met). This polymorphism affects the activity-dependent secretion of BDNF (Egan et al. 2003). Although at first, a negative association between the val66met polymorphism and hippocampal volume has been reported (Frodl et al. 2007), this observation was not supported by a meta-analysis that did not evidence any association between hippocampal volume and the val66met genotype in neuropsychiatric patients, perhaps because of the too many different disorders analyzed in this study (Harrisberger et al. 2015). Yet again, several other meta-analyses did confirm the association between the val66met polymorphism and an increased susceptibility to mood disorders (Hosang et al. 2014; Li et al. 2016; Yan et al. 2014; Zou et al. 2010). Finally, a recent study showed that subjects with the Met allele of the *Bdnf* gene had an increased risk for depression (Youssef et al. 2018). In this study, post-mortem analyses showed that depressed patients also have lower BDNF levels in the anterior cingulate cortex (ACC) and the caudal brainstem compared to non-depressed subjects, providing further evidence implicating low brain BDNF and the BDNF Met allele in major depression risk (Youssef et al. 2018).

#### BDNF and antidepressant treatments

The effect of antidepressants on BDNF expression and the role of BDNF in the treatment of MDD have been widely studied (see Castren and Kojima 2017 for a review). Interestingly, it has been shown that BDNF expression was increased in post-mortem brains of depressed patients treated with antidepressant drugs as compared to non-treated patients (Chen et al. 2001; Dunham et al. 2009).

The involvement of BDNF in the efficacy of antidepressant treatments has been demonstrated mainly in rodent models. Lee et al. developed a val66met mouse analogue, i.e., BDNF^met/met^ mice (Chen et al. 2006), and subsequent experiments showed that this genetic variant decreased fluoxetine efficacy through impaired synaptic plasticity defined by a reduction of theta-burst stimulation-induced LTP as well as impairments in the survival of newborn cells in the hippocampus (Bath et al. 2012; Ninan et al. 2010) and a deficit in synaptic transmission in the PFC (Pattwell et al. 2012). In addition, BDNF expression in the brain of rats was upregulated after chronic antidepressant drug exposure and ECT (Altar et al. 2003; Balu et al. 2008; Jacobsen and Mork 2004; Nibuya et al. 1995). Interestingly, some studies have shown that antidepressant-like effects observed in mouse models of depression after chronic administration of antidepressants were reversed by TrkB antagonist injection (Boulle et al. 2016; Ma et al. 2016; Yasuda et al. 2014) and that inhibition of TrkB signaling blocked the effects of antidepressants (Saarelainen et al. 2003). Interestingly, in addition to its effects on BDNF expression, autophosphorylation of TrkB by antidepressants has also been reported by the group of Castren (Rantamaki et al. 2007; Saarelainen et al. 2003) suggesting that TrkB can be transactivated independently from neurotrophins, as demonstrated in the case of glucocorticoid receptors (Jeanneteau et al. 2008). Indeed, a study by Rantamaki et al. (2011) showed a rapid action of imipramine on TrkB phosphorylation in either presence or absence of BDNF, suggesting that antidepressants do not require BDNF to activate TrkB, an effect that was independent of 5-HTT. These results highlight the potential direct effect of the BDNF-TrkB signaling pathway in the mechanism of action of antidepressants (Autry and Monteggia 2012) and evidenced that TrkB is a valuable target to treat depression.

#### Brain region-specific BDNF effects

In rodents, direct infusion of BDNF in the hippocampus (Deltheil et al. 2008, 2009; Shirayama et al. 2002; Sirianni et al. 2010) and midbrain (Siuciak et al. 1997) showed antidepressant-like effects. In contrast, an opposite, pro-depressive effect was reported after infusion of BDNF in the VTA or the NAc (Eisch et al. 2003). The same disparity was present using region-specific knockdown of BDNF expression. Impairment of BDNF signaling in the DG of the hippocampus (Taliaz et al. 2010) elicited pro-depressive behavior, whereas knockdown of BDNF in the NAc had an antidepressive effect (Berton et al. 2006). Interestingly, conditional knockout in the forebrain resulted in an increase in depressive-like behavior in female but not male mice (Monteggia et al. 2007) and, furthermore, decreased the efficacy of the antidepressant desipramine (Monteggia et al. 2004). This conditional knockout of BDNF in the forebrain displayed the same sex-specific incongruity in stress-induced depressive-like behavior (Autry et al. 2009). A different study using adeno-associated viral-mediated knockout of BDNF in the DG and CA1-region of the hippocampus showed that a loss of BDNF function in the hippocampus attenuated antidepressant drug treatment efficacy (Adachi et al. 2008).

Taken together, these data suggest that BDNF could be considered as a key therapeutic molecule against depression (Allen et al. 2015).

### Fibroblast growth factor or FGF

#### General function

The fibroblast growth factor (FGF) family has been described as a major player in proliferation and maturation of neurons in the SVZ and SGZ of the hippocampal DG (Woodbury and Ikezu 2014). The FGF family is composed of 18 ligands and 4 subtypes of receptors. FGF1 is expressed mostly in neurons while FGF2 is expressed in both neurons and glial cells. FGF1 and FGF2 are the most studied ligands of this family and have been shown to be dysregulated in mood disorders (Turner et al. 2006; Turner et al. 2012). They can bind all four receptor subtypes in order to activate the phospholipase C-γ (PLC-γ1 MAPK and AKT pathways (Turner et al. 2006; Turner et al. 2012). In addition, FGF1 and FGF2 were evidenced to play a critical role in the regulation of synaptic plasticity (Di Liberto et al. 2014).

#### FGF in neuroplasticity

Intracerebroventricular (ICV) injection of FGF2 induced neurogenesis in both the SVZ and SGZ (Jin et al. 2003; Mudo et al. 2009; Rai et al. 2007). Mice with a complete loss-of-function FGF2 allele showed a significant decrease in newly generated neurons but no reduction in proliferating cells (Werner et al. 2011). The additional increase in cell death in the hippocampus indicated a faulty neurogenesis following FGF2 knockout (Werner et al. 2011). Conditional knockout experiments with FGF receptor 1 (FGFR1)-*null* mice show defective LTP and neurogenesis (Zhao et al. 2007), suggesting that the FGF2-FGFR1 interaction might represent an important mediator of neurogenesis.

#### FGF in MDD

Post-mortem brain analysis in humans revealed a lower expression of FGF1 and FGF2 in the dorsolateral PFC and the ACC of patients with MDD (Evans et al. 2004). In addition, FGF2 was decreased in the hippocampus of depressed patients, whereas FGFR1 was increased (Gaughran et al. 2006). In rodents, Turner et al. reported reduced mRNA expression of FGF2 and its main receptor FGFR1 in the CA1, CA2, CA3, and DG following social defeat stress, a well-established model of depression (Turner et al. 2008a). Injection of FGF2 was also shown to reduce depressive-like behavior in rats (Turner et al. 2008b). A more recent study also reported that increased cell proliferation in the PFC following FGF2 infusions might also be involved in the antidepressant actions of FGF2 (Elsayed et al. 2012).

### Vascular endothelial growth factor or VEGF

#### General function

Vascular endothelial growth factor (VEGF) is primarily known for its induction of angiogenesis and modulation of vascular permeability during embryogenesis and growth, as well as pathological events such as in tumorigenesis. It can bind to different receptors: receptor tyrosine kinases (VEGFR) 1 and 2 with a higher affinity for VEGFR1. In addition, mounting evidence suggests that VEGF can be considered as a potent neurotrophic factor, inducing neurogenesis, neuronal survival and proliferation, glia survival, and glia migration (Carmeliet and Ruiz de Almodovar 2013).

#### VEGF in neuroplasticity

Interestingly, experimental studies showed that VEGF displays robust neuroprotective effects in cell models of ischemia and hypoxia (Jin et al. 2000) as well as a positive effect on neuronal growth, maturation, and proliferation under normoxic conditions (Khaibullina et al. 2004; Rosenstein et al. 2003; Silverman et al. 1999; Sondell et al. 1999; Zhu et al. 2003). A role in the development of dendrites and axons has also been described for VEGF (Khaibullina et al. 2004; Licht et al. 2010; Rosenstein et al. 2003; Sondell et al. 1999). Moreover, ICV administration of VEGF increased neuroprotection and neurogenesis in the adult rat brain after ischemia (Sun et al. 2003). More specifically, ICV administration of VEGF increased neurogenesis in both the SVZ and the SGZ of the DG with enhanced proliferation of neurons, astroglia, and endothelial cells (Jin et al. 2002), while VEGF-B knockout mice showed impaired neurogenesis (Sun et al. 2006). Hence, the overexpression of VEGF in the hippocampus using an adeno-associated viral vector in rats resulted in increased neurogenesis and was associated with improved learning and memory (Cao et al. 2004; During and Cao 2006). Interestingly, VEGF can promote neurogenesis by stimulating endothelial cells in order to release other neurotrophic factors (Yamada 2016). In addition, ependymal cells can synthesize VEGF leading to a stimulation of the VEGFR2 and inducing proliferation of neuronal precursors and enhanced formation of new neurons in the hippocampus (Nowacka and Obuchowicz 2012).

#### VEGF in MDD

A multitude of studies have investigated the plasma concentration of VEGF in MDD patients, but the data and interpretation remain conflicting possibly due to the differences in study designs (see review by Clark-Raymond and Halaris 2013) In addition, as mentioned above, there is currently no evidence that blood levels of neurotrophins reflect those of the brain, making causal inferences of the role of VEGF in MDD based on blood concentrations rather speculative. Nevertheless, angiogenesis seemed to be mediating the SSRI-induced upregulation of neurogenesis through VEGF (Yamada 2016). Indeed, higher hippocampal angiogenesis and neurogenesis have been found in SSRI-treated MDD patients when compared with untreated or healthy individuals (Boldrini et al. 2012). Chronic stress in rats decreased the expression of VEGF and its receptor in the hippocampus (Heine et al. 2005). Furthermore, VEGF is required for the proliferation of neural stem-like cells in the hippocampus following ECT treatment (Elfving and Wegener 2012; Segi-Nishida et al. 2008). In a similar manner, VEGF also seems to be required for the behavioral action of various antidepressant drugs in rodent models of depression (Greene et al. 2009; Sun et al. 2012; Warner-Schmidt and Duman 2007; Warner-Schmidt and Duman 2008).

### Glial cell line-derived neurotrophic factor or GDNF

#### General function

Glial cell line-derived neurotrophic factor (GDNF) was first discovered in a glial cell line but is expressed in many brain regions. It is a member of the transforming growth factor β (TGF-β) superfamily and is important for neuronal survival especially for dopaminergic and serotonergic neurons. It binds to the GDNF-family receptors α1 (GFRα1) activating tyrosine kinase signaling (Sharma et al. 2016).

#### GDNF in neuroplasticity

Experimental studies in animal models evidenced a neuroprotective role of GDNF, and ICV infusion of GDNF increased progenitor cell proliferation in the DG (Dempsey et al. 2003) and SVZ (Kobayashi et al. 2006). Similarly, infusion of GDNF in the striatum of rats increased progenitor cell proliferation in the hippocampus and substantia nigra (Chen et al. 2005). Moreover, GDNF induced differentiation of DG-derived neural precursors into astrocytes in vitro (Boku et al. 2013). Further, the use of an adeno-associated viral vector that induced overexpression of GDNF in the rat cortex provided neuroprotection against ischemia-induced injury (Tsai et al. 2000).

#### GDNF in MDD

Only a few clinical studies examined the role of GDNF in MDD, and contrasting findings between brain and blood expression are reported (Sharma et al. 2016). A recent post-mortem brain analysis showed an increase of (GDNF) expression in the parietal cortex of MDD patients (Michel et al. 2008). To our knowledge, this is the only region that has been studied in humans with respect to GDNF, although several studies have investigated the serum level of GDNF in MDD (Diniz et al. 2012a, b; Pallavi et al. 2013; Zhang et al. 2008) and under antidepressant treatment (Zhang et al. 2008).

In addition, antidepressant exposure increased GDNF release in a rat C6 glioblastoma cell line (Hisaoka et al. 2001), whereas lithium treatment in rats resulted in increased GDNF concentrations in the PFC and occipital cortex but a decrease in the hippocampus (Angelucci et al. 2003b). In mice exposed to chronic ultra-mild stress, an increase in *Gdnf* mRNA expression was observed in the hippocampus. This modification was partly reversed by chronic administration of the antidepressant agomelatine (Boulle et al. 2014). Furthermore, work on the same mouse model showed that GDNF, with other neurotrophins, could be involved in mediating the behavioral response to antidepressants (Uchida et al. 2011). Thus far, the exact involvement of GDNF in the etiology of depression is not fully understood, but its neuroprotective capacity might make it an interesting future target for antidepressant treatment.

### Insulin-like growth factor or IGF-1

#### General function

Insulin-like growth factor (IGF-1) and its receptor IGF-1R are found in many tissues including the brain (Aberg et al. 2006). It influences growth and differentiation processes (Frysak et al. 2015). IGF-1 has been designated as a potential therapeutic target for neurodegenerative diseases such as MDD (Szczesny et al. 2013).

#### IGF-1 in neuroplasticity

IGF-1 induced differentiation of neuronal precursors (Anderson et al. 2002; Arsenijevic and Weiss 1998) and proved to be neuroprotective in cerebellar granule neurons in vitro (D’Mello et al. 1993). IGF-1 knockout mice showed a decrease in total brain size and SGZ volume, further supporting the importance of IGF-1 in neurodevelopment (Beck et al. 1995). Developmental research in mice has revealed the importance of IGF-1 in hippocampal neurogenesis and synaptogenesis (O’Kusky et al. 2000). Furthermore, ICV infusion of IGF-1 ameliorated the age-related decline in hippocampal neurogenesis (Lichtenwalner et al. 2001), while peripheral administration of this growth factor could induce hippocampal neurogenesis in rats (Åberg et al. 2000).

#### IGF-1 in MDD

Although clinical studies showed somewhat inconsistent findings, they however mainly revealed higher IGF-1 levels in the serum of depressed patients, which declined during effective antidepressant treatment (Bot et al. 2016; Kopczak et al. 2015; Szczesny et al. 2013). In contrast, IGF-1 was high in cerebrospinal fluid of antidepressant-treated patients (Schilling et al. 2011). These data suggest differential actions of IGF-1 in the periphery and the brain.

However, preclinical studies using conditional knockout mice showed that a decrease in either systemic or hippocampal IGF-1 levels could increase the susceptibility to develop depressive-like behavior (Mitschelen et al. 2011). In addition, in corticosterone-treated rats, a decrease of IGF-1 in both the hippocampus and serum has been reported. However, physical exercise using continuous running was not able to reverse this diminution while it did prevent depressive-like behavior (Yau et al. 2014). Interestingly, since IGF-1 can readily pass the blood-brain barrier (Pan and Kastin 2000) in contrast to BDNF, its effects on the brain can be achieved by direct injection into the blood. In line with this notion, when administrated chronically, a peripheral injection of IGF-1 in a mouse model of depression was shown to induce antidepressant-like behaviors, comparable to commonly used antidepressants (Duman et al. 2009). In addition, an increase of peripheral IGF-1 by direct injection of IGF-1 or inhibition of IGF-1 binding protein displayed anxiolytic and antidepressant effects in rodents (Duman et al. 2009; Hoshaw et al. 2005; Malberg et al. 2007; Park et al. 2011), which might be attributed, at least in part, to increased serotonin levels in the brain (Hoshaw et al. 2008). Of note, intranasal administration has been proposed in order to provide a shorter path for IGF-1 to enter the brain (Paslakis et al. 2012), avoiding unwanted effects of IGF-1 in peripheral tissues.

### Nerve growth factor or NGF

#### General function

Nerve growth factor (NGF) is a growth factor first described as a neurite outgrowth factor (Olson 1967). Later, NGF proved to be involved in neuronal repair and survival (Kromer 1987; Shigeno et al. 1991; Sofroniew et al. 2001; Zhao et al. 2004). NGF has been implicated in proliferation and differentiation of neuronal stem cells (Cattaneo and McKay 1990) and more recently in neurogenesis in the striatum (Frielingsdorf et al. 2007; Zhu et al. 2011) and in regulating hippocampal plasticity (Conner et al. 2009).

#### NGF in MDD

To date, only a few studies have investigated the role of this trophic factor in MDD and only at peripheral level.

In the Flinders Sensitive Line (FSL) rat model of depression, ECT was found to increase NGF levels in the hippocampus (Angelucci et al. 2003a). In addition, subcutaneous NGF injections show antidepressant effects (Overstreet et al. 2010).

**Fig. 3:**
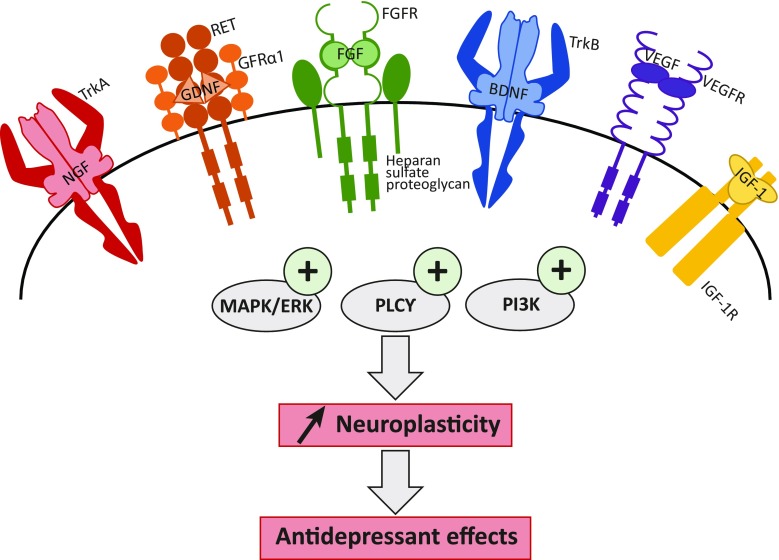
Neurotrophins increase neuroplasticity through the activation of three main signaling pathways. Neurotrophins bind to their receptors in order to promote three main signaling pathways: the MAPK/ERK, the PI3-K, and the PLCγ signaling cascades. Once activated, they stimulate neuroplasticity, especially synaptic plasticity, neurotransmission and neuronal survival, growth, and differentiation. An increase of neuroplasticity is likely to induce antidepressant effects. BDNF, brain-derived neurotrophic factor; TrkB, tropomyosin-related kinase receptor B; FGF, fibroblast growth factor; FGFR, fibroblast growth factor receptor; VEGF, vascular endothelial growth factor; VEGFR, vascular endothelial growth factor receptor; GDNF, glial cell line-derived neurotrophic factor; GFRα1, GDNF-family receptor-α; IGF-1, insulin-like growth factor 1; IGF-1R, insulin-like growth factor 1 receptor; NGF, nerve growth factor; TrkA, tropomyosin-related kinase receptor A

This—non-exhaustive—review of the literature further underlines the potential effects of the different members of the neurotrophin family in the alteration of neuroplasticity often observed in depression-like disorders, as summarized in Fig. 3.

## Future directions: targeting growth factor signaling with synthetic small molecules, focus on the BDNF/TrkB pathway

Over the last decades, convergent studies suggested that the BDNF/TrkB signaling pathway was a main actor in the development and course of mood disorders, in particular depression, as well as in the action of currently available antidepressants. Because of the poor efficacy of antidepressants and their delayed therapeutic effect, there is a need to find novel and more efficacious compounds. The use of BDNF itself turned out to be rather difficult because of its unfavorable pharmacokinetic profile. Indeed, peripheral administered BDNF hardly crosses the blood-brain barrier and has a very short half-life. Interestingly, the recent data suggesting that antidepressant could also directly transactivated TrkB (Rantamaki et al. 2011) point to this receptor as a main molecular target for treating depression. Thus, attempts to develop new molecules that directly target TrkB signaling have been undertaken as proposed by Tsai (2007).

### TrkB agonists

#### 7,8-Dihydroxyflavone

The most extensive examined TrkB agonist so far is 7,8-dihydroxyflavone (DHF). It was first described as an antioxidant and later identified as a TrkB agonist after a screening of flavone derivatives (Choi et al. 2010, Liu et al. 2010). Many experiments have been performed and have revealed promising results regarding antidepressant-like properties. Recent studies show that DHF has antidepressant effects in mice displaying LPS-induced depressive-like behavior in the tail suspension test (TST) and the forced swim test (FST) (Zhang et al. 2014a). In the learned helplessness model of depression in rats, a single bilateral infusion of DHF in various subregions of the hippocampus and in the infralimbic medial PFC induced antidepressant effects (Shirayama et al. 2015).

Furthermore, DHF has been shown to normalize dendritic spine structure in an LPS model of depression (Zhang et al. 2014a) and to increase neurogenesis in the hippocampus of naïve mice (Liu et al. 2010). Besides its effect on depressive-like behavior, further studies have tested DHF on cognition. Indeed, restoration of memory deficits in 5XFAD and APP/PS1 mouse models of Alzheimer’s disease (AD) have been shown after both acute and chronic administration of DHF (Bollen et al. 2013; Devi and Ohno 2012; Zhang et al. 2014c), while it did not seem to exert this effect when injected chronically in APP23PS45 transgenic mice (Zhou et al. 2015). Because memory impairments were observed in MDD patients (Dere et al. 2010; Millan et al. 2012), the recovery of memory deficits in animal models of AD after administration of DHF could be expected. Liu et al. optimized the molecule by synthesizing various bioisosteric derivatives and showed an even more pronounced antidepressant effect in both the FST and TST requiring a lower dose for a more potent effect (Liu et al. 2010; Liu et al. 2012).

#### Other promising agonists

*Gedunin* is a tetranortriterpenoid isolated from the Indian neem tree. Its derivative, deoxygedunin, seems to be a promising selective TrkB agonist, showing protective effects against apoptosis, both in vitro, in hippocampal neurons incubated with a toxic dose of DMSO, and in vivo, in mice that received kainic acid (Jang et al. 2010). Antidepressant effects of deoxygedunin have also been shown after subchronic treatment, displaying reduced immobility in the FST (Jang et al. 2010).

*LM22A-4* was designed to mimic the loop II domain of BDNF and proven to be a partial agonist. In vitro work showed that LM22A-4 has neuroprotective properties (Massa et al. 2010). It was first shown as an effective molecule to reverse respiratory abnormalities (Kron et al. 2014; Schmid et al. 2012) and to improve functional recovery after stroke (Han et al. 2012). Furthermore, it was also shown to reverse alcohol drinking in mice.

*TDP6* and *29D7* have been shown to represent other promising BDNF mimetic molecules able to promote oligodendrocyte-mediated myelination in vitro (Wong et al. 2014) and to enhance neuronal survival and neurotic outgrowth in vitro and in vivo as well as to provide long-lasting neuroprotection against neonatal hypoxic-ischemic brain injury (Kim et al. 2014; Qian et al. 2006).

*TAM-163*, an antibody targeting TrkB, has been shown to be a partial TrkB agonist but only tested as an agonist agent for body weight regulatory disorders (Perreault et al. 2013).

Another way to activate Trk receptors is to potentiate their neurotrophic-mediated activation. *BMS355349* has been described as a selective potentiator of NT-3 mediated TrkA and TrkB receptor activity and has proven to induce neurogenesis (Chen et al. 2009; Lewis et al. 2006).

To sum up, further investigations are required regarding the action of TrkB agonists in mood disorders, but their observed neuroprotective effects so far are promising.

### TrkB antagonists

Knowing that, in a rat model of depression, increases in BDNF in the VTA-NAc (Shirayama et al. 2015; Zhang et al. 2015b) induced pro-depressive-like effects (Eisch et al. 2003), the use of partial antagonists as a treatment for diseases related to nervous system dysregulations has been considered. To our knowledge, only ANA-12 and cyclotraxin B, both described as selective antagonists for TrkB, have been tested so far.

*ANA-12* is a selective partial agonist that was first developed by Carzola et al. in 2011 using a structure-based in silico screening (Cazorla et al. 2011). In their study, the authors identified a low-molecular weight antagonist of TrkB that could induce anxiolytic and antidepressant properties. While ANA-12 was mainly used as a tool to block the BDNF/TrkB complex in order to better understand the mechanism of action of TrkB signaling (Montalbano et al. 2013), two recent studies tried to use it as a therapeutic agent. When infused bilaterally in the NAc, ANA-12 showed antidepressant properties in the TST and FST in LPS-treated mice (Zhang et al. 2014a). Moreover, in mice that exhibit a reduced social interaction, ANA-12 infusion into the NAc has been shown to completely block social avoidance (Walsh et al. 2014). When administrated intraperitoneally and alone, ANA-12 could also decrease the immobility time in the TST and the FST in the same LPS-treated mice.

*Cyclotraxin B* has been mainly used to antagonize BDNF/TrkB signaling. Nevertheless, some behavioral studies have been performed. While one study has observed anxiolytic properties, this molecule did not seem to display clear antidepressant effects (Cazorla et al. 2010) but it may be suitable for the treatment of neuropathic pain (M’Dahoma et al. 2015). In spite of these interesting results, antagonists could also induce cell death, an issue that should be addressed when considering using these agents in chronic treatment regimens (Cazorla et al. 2011; Takemoto et al. 2015).

It is often observed that in the presence of the endogenous ligand, a synthetic molecule presents antagonistic properties. This was, e.g., shown in a study where they screened several potential TrkB agonists in cells stably transfected with TrkB. When the cells were solely incubated with the synthetic molecules, an increase of TrkB phosphorylation was observed. However, in presence of BDNF, the molecules lost their agonistic effect and showed an antagonistic effect with a decrease of P-TrkB (Cardenas-Aguayo Mdel et al. 2013). Thus, the use of partial agonists or antagonists in order to treat mood disorders seems to be a promising avenue of research. However, whether we should agonize or antagonize the pathway is not yet well defined. Also, further investigations have to be achieved regarding the role of the BDNF/TrkB complex in mood disorders, in order to have a better knowledge of how to correct the dysregulation of this system in mental illness.

## Concluding remarks

Growth factors and associated neurotrophic signaling play an essential role in the development and maintenance of the central nervous system (Anlar et al. 1999; Ford-Perriss et al. 2001; Greenberg et al. 2009). Accordingly, there is growing evidence that abnormal trophic support in cortico-limbic regions regulating mood and emotions may take part in the pathophysiology of depression (Krishnan and Nestler 2008). In addition, clean-cut evidence shows that antidepressants require neuroplasticity pathways to rescue the observed deficits in neuronal and synaptic plasticity often associated with mood disorders (Pittenger and Duman 2008). However, current knowledge makes it difficult to conclude whether neuroplasticity and neurogenesis represent a cause, a consequence (or both), or an epiphenomenon of the pathological processes associated with depression. Hence, future research should focus on elucidating the exact involvement of neurotrophic signaling in the onset and course of major depression. Furthermore, mounting evidence seems to indicate that neurogenesis might not be required for the therapeutic action of antidepressants (Bessa et al. 2009). In line with this hypothesis, the usage of *N*-methyl-d-aspartate (NMDA) receptor antagonists showed that acute induction of neuroplasticity pathways, e.g., increased BDNF signaling, was sufficient to produce a robust and prolonged antidepressant effect (Autry et al. 2011; Li et al. 2010). Hence, the rapid enhancement of hippocampal neuroplasticity—involving dendritic growth, spine density, and synaptic transmission—represents an original strategy to circumvent the delayed efficacy of current antidepressant drugs. Finally, the use of drugs that specifically target neurotrophic signaling should provide more insights in the role of neuroplasticity pathways in antidepressant responses. As growth factors and neurotrophins generally display a poor blood-brain barrier penetration and a short half-life in plasma (Nave et al. 1985; Ochs et al. 2000; Poduslo and Curran 1996), identification of small non-peptidic neurotrophin mimetics, albeit challenging on its own, may represent an interesting target for the development of a new class of therapeutic agents for mood-related disorders.
